# The Experiences in a Toxicology Unit: A Review of 623 Cases

**DOI:** 10.4021/jocmr1687w

**Published:** 2013-12-13

**Authors:** Ramazan Koylu, Zerrin Defne Dundar, Oznur Koylu, Emine Akinci, Nazire Belgin Akilli, Mustafa Onder Gonen, Basar Cander

**Affiliations:** a Konya Training and Research Hospital, Emergency Department, Konya, Turkey; b Konya Training and Research Hospital, Biochemistry Department, Konya, Turkey; c Necmettin Erbakan University Meram Faculty of Medicine, Emergency Medicine Department, Konya, Turkey

**Keywords:** Toxicology, Poisoning, Epidemiology, Suicide

## Abstract

**Background:**

To evaluate the etiological and demographic characteristics of adult poisoning patients followed up in a toxicology unit in Konya, Turkey.

**Methods:**

Patients (≥ 15 years old) followed up with the diagnosis of poisoning in our toxicology unit in 2011 were included in this retrospective study. The patients’ medical records were investigated. Age, gender, medical history, the first medical center the patient had been admitted to, the routes and causes of poisoning, the toxins involved, the number of the pills taken, treatments, complications, the length of stay in the hospital and the outcome were recorded.

**Results:**

A total of 623 patients were included in the study. The mean age of patients was 28.1 ± 15.1. Four hundred and forty-five (71.4%) of patients were female, 541 (86.9%) of them were poisoned via the oral route and 75 (12.0%) of them were poisoned by inhalation. The causes of poisoning were drugs in 408 (65.5%) patients, pesticides/insecticides in 58 (9.3%) patients and carbon monoxide in 49 (7.9%) patients. The commonly used drugs were as follows: analgesics (57.2%), antidepressants (25.4%) and gastrointestinal system drugs (15.8%). The poisonings were suicidal in 489 (78.5%) patients, accidental in 120 (19.3%) patients and overdose in 14 (2.2%) patients. The number of women was higher in the suicide group. At the end of the treatment, 604 (97.0%) of the patients were discharged and 3 (0.4%) of them died. The duration of follow-up was 39.2 ± 37.5 h.

**Conclusion:**

The most common causes of poisoning are drugs, pesticides/insecticides and carbon monoxide. Health and educational policies at a national level are needed in order to prevent this medicosocial problem. Furthermore, specially equipped toxicology units should be constructed for the treatment and follow-up of the poisoned patients in order to reduce the morbidity and mortality to a significant extent.

## Introduction

Suicidal and accidental poisonings are important and frequent reasons for admissions to the emergency units. The rate of poisoning in USA was reported to be 479/100,000 in 2011 and the rate of poisoning-related deaths was reported to be 17/100,000 in 2010 [1, 2]. In contrast to the previous years, poisoning has become the most frequent reason for accidental deaths with a rate surpassing motor vehicle-related mortalities [3]. Different morbidity and mortality rates due to poisoning have been reported worldwide [4-7].

The etiological and demographic characteristics of poisoning cases differ even in different geographical regions of the same country. Various outcomes have been reported in studies conducted in different regions of our country as well. In general, the poisonings in our country are suicidal and are common amongst women. However, pesticides are important causes of poisoning in farming regions, and carbon monoxide and fungi are important causes of poisoning in some other regions [8-12].

The aim of this retrospective study performed in 2011 was to present the etiological and demographic characteristics of poisoned adult patients followed up in the toxicology unit of the emergency department, in a training and research hospital in Konya, a city of the Middle Anatolian Region of Turkey.

## Methods

Patients (≥ 15 years and older) who were followed up with the diagnosis of poisoning in our toxicology unit in 2011, were included in this retrospective study. The patients’ medical records were investigated. Age, gender, medical history, the first medical center the patient had been admitted to, the route and cause of poisoning, the toxins, the number of the pills taken, treatments, complications, the length of stay in the hospital and the outcome were recorded.

Our toxicology unit began functioning under the operation of the emergency unit in March 2011 with a 6-bed capacity. Every bed is equipped with a bedside unit including a monitor, a stationary aspirator, an oxygen source and a medical air source compatible with a ventilator. There are two mechanical ventilators and one bedside hemodialysis/hemoperfusion instrument in the toxicology unit. The multi-drug test could not be performed due to some technical issues during the study, but the levels of the drugs were measured in the central biochemistry laboratory of our hospital throughout the study. Therefore, the multi-drug detection of the patients could not be investigated.

The toxins involved in our study were divided into 8 groups including drugs, pesticides/insecticides, rodenticides, carbon monoxide, foods, corrosives, others (narcotics, alcohol, and so on) and unknowns. The drugs were also divided into 12 groups including analgesics, antibiotics, antihistamines, decongestants, antidepressants, other psychoactive drugs (antipsychotics, benzodiazepines, barbiturates, and so on), anticonvulsants, antidiabetics, cardiovascular system drugs, gastrointestinal system drugs, others (vitamins, hormones, and so on) and unknowns. The patients were divided into 3 groups according to the cause of poisoning (suicidal, accidental and overdose).

The descriptive statistics were performed using the SPSS version 16.0 (SPSS Inc., Chicago, USA). The data were expressed as frequencies, percentages and mean ± SD. The categorical data were compared using the chi-square test and the constant data were compared using the Student’s t test or the Mann-Whitney U test. A P value of < 0.05 was evaluated as statistically significant.

## Results

**Figure 1:**
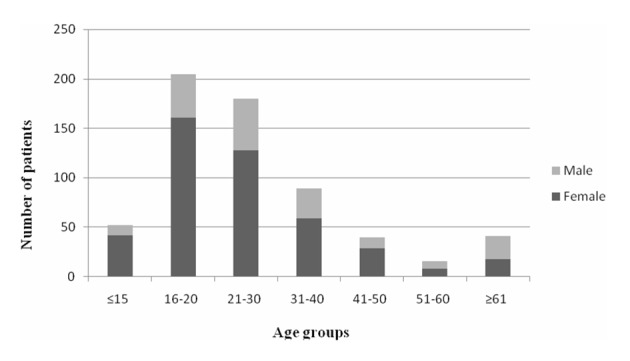
The gender distribution of the patients with regard to the age groups.

**Figure 2:**
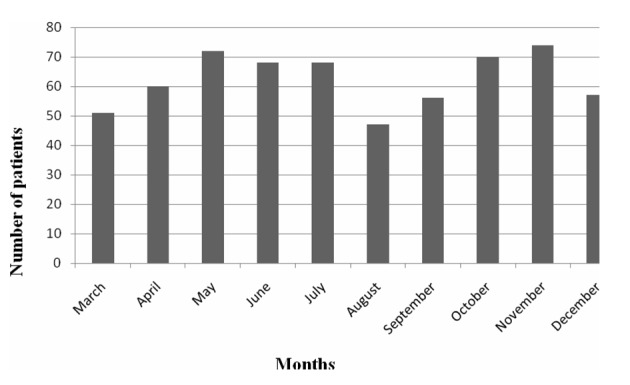
The distribution of poisoning cases according to months.

The medical charts of 641 patients who were followed up in our toxicology unit in 2011 were retrospectively investigated. Eleven patients with missing information and 7 patients who were determined not have been poisoning cases were excluded from the study. A total of 623 patients that fulfilled the inclusion criteria were included in the study. One hundred and seventy-eight (28.6%) of patients were male and 445 (71.4%) of them were female. The mean age was 28.1 ± 15.1 (range 13 - 90). The 16 - 20 age group included the maximum number of cases, which was 205 (32.9%). The number of female patients was statistically significantly higher than the number of male patients in all groups, except for the 51 - 60 and the ≥ 61 age groups (P < 0.001). Figure 1 shows the age groups and the gender distribution of the patients. The distribution of poisoning cases within months was not different (Fig. 2).

Six hundred and four (97.0%) of patients did not have a history of additional disease, while 12 (1.9%) of them had at least one systemic disease such as diabetes mellitus or hypertension. Seven (1.1%) patients had various psychiatric disorders. Fifteen (3.4%) women were pregnant. Four hundred and five (65.0%) of patients had presented to our emergency unit directly, while the other 218 (35.0%) patients had been referred from other medical centers.

Among 623 patients, 541 (86.9%) of them were poisoned via the oral route, 75 (12.0%) of them were poisoned by inhalation, 5 (0.8%) of them were poisoned dermally, and 2 (0.3%) of them were poisoned via the parenteral route. The poisoning agents were drugs in 408 (65.5%) patients, pesticides/insecticides in 58 (9.3%) patients, carbon monoxide in 49 (7.9%) patients, foods in 28 (4.5%) patients, rodenticides in 12 (1.9%) patients, corrosives in 3 (0.5%) patients, and other substances such as alcohol or narcotics in 13 (2.1%) patients. Multiple substances from several groups were the poisoning agents in 14 (2.2%) patients. Thirty-eight (6.1%) patients had stated that they had been poisoned via the oral route, but no anamnesis could be obtained regarding the substance of poisoning.

According to the number of exposed active substances, the poisoning occurred due to 1 active substance in 373 (59.9%) patients, 2 substances in 86 (13.8%) patients, 3 substances in 55 (8.8%) patients, 4 substances in 27 (4.3%) patients, 5 substances in 11 (1.8%) patients, and 6 or more substances in 9 (1.4%) patients. The active substances in 62 (10.0%) patients could not be identified.

**Figure 3:**
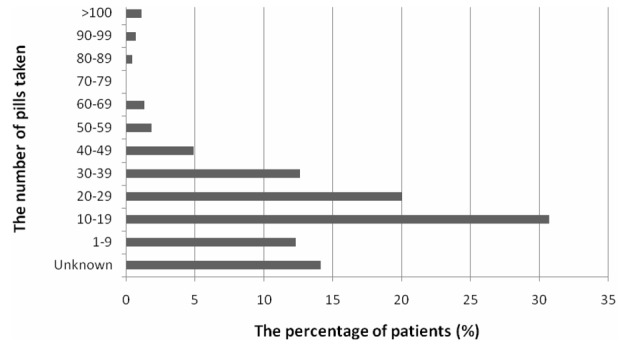
The total number of pills taken.

The drug groups included in the poisoning of 418 patients have been presented in Table 1. Fifty-one (8.2%) of patients had been poisoned by preparations containing two or more active substances, which were anti-flu drugs (different combinations of paracetamol, decongestants, antihistamines and antitussives) in 46 (7.4%) patients, anti-migraine drugs in 4 (0.6%) patients, and anti-ulcer drugs in 2 (0.3%) patients. The percentages were calculated considering a total of 418 patients. The sum of percentages was over 100, since there were patients poisoned with multiple drugs. The total number of pills used by 446 (71.6%) patients in the drug-related poisoning group has been displayed in Figure 3.

**Table 1:** Drug Groups Involved in the Poisoning of 418 Patients

	n (%)*
Analgesics	239 (57.2)
Antidepressants	106 (25.4)
Gastrointestinal system drugs	66 (15.8)
Antihistamines	60 (13.4)
Antibiotics	54 (12.9)
Decongestants	49 (11.7)
Cardiovascular system drugs	37 (8.9)
Other pyschoactive drugs	36 (8.6)
Anticonvulsants	18 (4.3)
Antidiabetics	16 (3.8)
Others	69 (16.5)
Unknown	24 (5.7)

The cause of poisoning was suicide in 489 (78.5%) of patients, accidental in 120 (19.3%) of them and overdose in 14 (2.2%) of them. The number of women in the suicidal poisoning group (375 cases) was statistically significantly higher than the number of men (114 cases) (P < 0.001). According to age groups, 38.9% of the patients belonged to the 16 - 20 age group among the suicidal poisoning group, and 31.9% belonged to the 21 - 30 age group (P < 0.001). No accumulation was present in a certain age group for the patients in accidental or overdose groups (P > 0.05). No significant difference was present between the lengths of stay in the hospital of the three groups (P > 0.05). The patient group properties with regard to the cause of poisoning have been presented in Table 2.

**Table 2:** Patient Group Properties According to the Type of Poisoning

	Suicidal (n = 489)	Accidental (n = 120)	Overdose drug use (n = 14)	Total (n = 623)
Gender				
Male	114 (23.3)	57 (47.5)	7 (50.0)	178 (28.6)
Female	375 (76.7)	63 (52.5)	7 (50.0)	445 (71.4)
Age groups				
≤ 15	44 (9.0)	6 (5.0)	2 (14.3)	52 (8.3)
16 - 20	190 (38.9)	12 (10.0)	3 (21.4)	205 (32.9)
21 - 30	156 (31.9)	22 (18.3)	2 (14.3)	180 (28.9)
31 - 40	65 (13.3)	23 (19.2)	1 (7.1)	89 (14.3)
41 - 50	21 (4.3)	19 (15.8)	0 (0.0)	40 (6.4)
51 - 60	4 (0.8)	12 (10.0)	0 (0.0)	16 (2.6)
≥ 61	9 (1.8)	26 (21.7)	6 (42.9)	41 (6.6)
Medical history				
No disease	478 (97.8)	118 (98.3)	8 (57.1)	604 (97.0)
Systemic disease	5 (1.0)	2 (1.7)	5 (35.7)	12 (1.9)
Psychiatric disease	6 (1.2)	0 (0.0)	1 (7.2)	7 (1.1)
First center of admission				
Other health centers	188 (38.4)	27 (22.5)	3 (21.4)	405 (65.0)
Our emergency unit	301 (61.6)	93 (77.5)	11 (78.6)	218 (35.0)
Route of poisoning				
Oral	488 (99.8)	43 (35.8)	10 (71.4)	541 (86.9)
Inhalation	0 (0.0)	72 (60.0)	3 (21.4)	75 (12.0)
Dermal	0 (0.0)	5 (4.2)	0 (0.0)	5 (0.8)
Parenteral	1 (0.2)	0 (0.0)	1 (7.1)	2 (0.3)
Toxin				
Drugs	398 (81.4)	2 (1.7)	8 (57.1)	408 (65.5)
Pesticide/insecticide	25 (5.1)	33 (27.5)	0 (0.0)	58 (9.3)
Carbon monoxide	0 (0.0)	49 (40.8)	0 (0.0)	49 (7.9)
Foods	0 (0.0)	28 (23.3)	0 (0.0)	28 (4.5)
Ronticides	10 (2.0)	2 (1.7)	0 (0.0)	12 (1.9)
Corrosives	2 (0.4)	1 (0.8)	0 (0.0)	3 (0.5)
Others	3 (0.6)	5 (4.2)	5 (35.8)	13 (2.1)
With more than one group	13 (2.7)	0 (0.0)	1 (7.1)	14 (2.2)
Unknown	38 (7.8)	0 (0.0)	0 (0.0)	38 (6.1)
Duration of hospital stay (hours)	38.7 ± 38.4	41.3 ± 35.1	39.6 ± 30.5	39.2 ± 37.5
Result				
Discharge	475 (97.1)	116 (96.7)	13 (92.9)	604 (97.0)
Transfer to normal room	11 (2.3)	4 (3.3)	1 (7.1)	16 (2.6)
Exitus	3 (0.6)	0 (0.0)	0 (0.0)	3 (0.4)

Gastric lavage was performed and activated charcoal was administered to 316 (50.7%) patients in our unit and to 192 (30.8%) patients in other health centers. None of the interventions were applied to 115 (18.4%) patients. Antidote was given to 196 (31.5%) of 623 patients. The antidote was N-acetylcysteine in 65 (10.4%) patients, oxygen in 48 (7.7%) patients, bicarbonate in 34 (5.4%) patients and pralidoxime (PAM) in 18 (2.9%) patients.

No complication was observed in 600 (96.3%) of patients, while 11 (1.8%) of them had rhabdomyolysis, 8 (1.3%) of them had acute kidney failure, 2 (0.3%) of them had seizures, 1 (0.2%) of them had arrhythmia and 1 (0.2%) of them had dystonia, 7 (1.1%) of patients underwent hemodialysis, 5 (0.8%) of them underwent hemoperfusion, and 1 (0.2%) of them patient underwent plasmapheresis. Four (0.6%) patients needed mechanical ventilation during the follow-up period, and 1 (0.2%) patient underwent emergency endoscopy.

Six hundred and four (97.0%) of patients were discharged from the toxicology unit at the end of the observational and treatment period, 16 (2.6%) patients were referred to other wards and 3 (0.4%) patients died. The mean observation period of the patients in the toxicology unit was 39.2 ± 37.5 (range 2 - 504) h.

## Discussion

Poisoning is currently an important health problem worldwide. It is a frequent cause of admissions to emergency units in our country as well. Herein, we share our experiences regarding the cases of poisonings we followed up in the toxicology unit.

In our study, 71.4% of all the patients were women. In almost all of the studies from different regions of our country, women have shown the higher percentage of poisoning, which ranges between 53% and 73% [8-14]. In other countries, this range is 49-78% [4-6, 15]. The mean age was 28 in our study and 61.8% of all the patients were in the 16 - 30 age group. Women in this age group reached a rate of 79%. It has been reported that the rates of suicidal poisoning are high, especially in younger women in developing countries with patriarchal family structures such as Turkey [8-12, 15-17].

It was observed that 78.5% of the poisoning cases were suicidal. Similar suicide attempt rates have been reported in different regions of our country, such as Adana, Kayseri and Istanbul [8, 9, 11], 46-62% suicide attempt rates have been reported in Pakistan and Norway, whereas this rate has been reported to be approximately 90% in India, Iran and Romania [4-6, 15-17]. This variation between the regions and countries may reflect different suicidal inclinations of different societies and their choice of suicidal attempt methods. This subject should be investigated by further studies.

The most common substances involved in poisoning cases in our study were drugs (65.5%), pesticides/insecticides (9.3%) and carbon monoxide (7.9%). Other studies from Turkey have reported the drugs as the most common poisoning agent as well [8-14]. Pesticide/insecticide poisonings are common in some regions of India and Sri Lanka, whereas alcohol is common in Norway and drugs are common in Romania, Iran and USA [4, 5, 7, 15, 18, 19]. This shows that the agent of poisoning may differ according to the regional and sociocultural characteristics of a certain geography. The prevalence of use of toxic substances and easy availability are factors that affect the type of the exposed toxins.

Approximately 41% of accidental poisonings in our study were related to carbon monoxide. Carbon monoxide poisoning rates in our country have been reported to range between 2.1% and 10.2% [8-12]. Despite the years having passed and the organized programs for the awareness of the society, carbon monoxide poisoning remains an important cause of morbidity and mortality in winter.

The patients in the drug-related poisoning group were poisoned most frequently by analgesics (57.2%) and antidepressants (25.4%). The same situation is valid for some studies that have been conducted in Turkey [8, 9, 11, 20], while some others have reported an opposite ranking [11, 13, 21]. The most frequent agent of poisoning in USA is analgesics, whereas benzodiazepine poisoning is common in countries such as Iran, India and Norway [5, 6, 17, 19]. Analgesics and antidepressants, which are easily accessible and cheaper than other drug groups, are the most preferred drugs for suicides in Turkey, and that is in accordance with the results of our study.

In our study, 59.9% of the patients were poisoned by one active agent, while 13.8% were poisoned by two. The management of these patients is comparatively easy since the drug action and interactions are predictable. For the remaining 17% of patients, who were poisoned by 3 or more active agents, a careful follow-up is needed with possible aggressive decisions when necessary. The subject of principal importance here is the 10% of patients in whom the agent of exposure could not be determined by anamnesis or clinical findings. In emergency conditions, it is known that blood or urine toxicology tests are not valuable for the follow-up of most of the patients. However, the measurement of detectable compounds in blood such as paracetamol, salicylate and lithium, or in urine such as benzodiazepine and tricyclic antidepressants is important in the follow-up of patients and regulation of treatment protocols [22]. We believe that urine drug detection tests are necessary in the evaluation of the 10% of patients in whom anamneses could not be obtained and in patients with suicidal drug use in whom the anamneses are unreliable. Absence of urinary drug detection test was a deficiency in our study.

Hemodialysis and hemoperfusion were performed on 2.1% of the patients. In a study conducted in India, 2.9% of the patients were reported to have required hemodialysis [6]. The poisoning statistics of 2010 in USA show that 0.4% of the hospitalized poisoning cases had undergone either hemodialysis or hemoperfusion treatments [19]. Recently, extracorporeal treatments are being successfully used in the management of poisonings. Thus, we believe that toxicology units should possess the proper equipment.

In our study, no complication was observed on the follow-up of 96.3% of the patients, and the mortality rate observed was 0.4%. Mortality rates that have been reported from different cities of Turkey are as follows: 2.6% in Adana, 0.7% in Istanbul, 5.8% in Sanliurfa, 1.6% in Kayseri and 0% in Ankara [8-11, 21]. The mortality rates from other countries have been reported as: 2.3% in Iran, 0.8% in Norway and 2.8% in India [5, 6, 17]. The high mortality rates observed in regions with more widespread pesticide/insecticide poisonings such as Adana and India, are as expected. In our region, poisonings are usually related to less toxic agents such as analgesics or antidepressants, and thus, our mortality rates are rather low. Furthermore, we believe that rapid transfer of the patients to the toxicology unit following the first stabilization decreased our morbidity and mortality rates.

All of the studies concerning poisoning cases show that poisoning is a serious medical problem. Although different etiological and demographic data are observed from different regions of a country or from different countries, the only subject of consensus is that the poisoned patient needs a special management algorithm beginning with admission to the emergency unit until their discharge. Therefore, special toxicology units with ample facilities should be constructed in order to provide aggressive medical support when needed and a careful follow-up. The health policies of developed countries which support the construction of such toxicology units should be followed up in developing countries such as Turkey as well.

### Conclusion

The most frequent group of poisoned patients comprised women in the 16 - 30 age group in our study. The suicidal poisoning cases were 78.5% of the cases. The most frequent causes of poisoning were drugs, pesticides/insecticides and carbon monoxide. Health and education policies at a national level are needed in order to prevent this medicosocial problem. Furthermore, specially equipped toxicology units should be constructed for the treatment and follow-up of poisoned patients in order to decrease the morbidity and mortality to a significant extent.

### Limitations

Since our study was retrospective, there were no data on some important information such as marital status, reasons for suicide attempts, and whether these attempts were reactional or not. Although our toxicology unit is unique in our region, poisoning cases are being followed up in different wards of other hospitals as well. Therefore, our study does not demonstrate the real frequency of poisoning cases in our region.
